# Early Lactate Clearance as a Determinant of Survival in Patients with Sepsis: Findings from a Low-resource Country

**DOI:** 10.2478/jccm-2023-0005

**Published:** 2023-02-08

**Authors:** Fazal Rehman, Saad Bin Zafar, Adil Aziz, Abdul Aziz, Pirbhat Shams Memon, Taymmia Ejaz, Summaira Aziz

**Affiliations:** 1 Department of Medicine, The Aga Khan University Hospital, Karachi, Pakistan; 2 Department of Community Health Sciences, The Aga Khan University Hospital, Karachi, Pakistan

**Keywords:** sepsis, mortality, lactate, outcomes

## Abstract

**Background:**

Single lactate measurements have been reported to have prognostic significance, however, there is a lack of data in local literature from Pakistan. This study was done to determine prognostic role of lactate clearance in sepsis patients being managed in our lower-middle income country.

**Methods:**

This prospective cohort study was conducted from September 2019-February 2020 at the Aga Khan University Hospital, Karachi. Patients were enrolled using consecutive sampling and categorized based on their lactate clearance status. Lactate clearance was defined as decrease by 10% or greater in repeat lactate from the initial measurement (or both initial and repeat levels <=2.0 mmol/L).

**Results:**

A total 198 patients were included in the study, 51% (101) were male. Multi-organ dysfunction was reported in 18.6% (37), 47.7% (94) had single organ dysfunction, and 33.8% (67) had no organ dysfunction. Around 83% (165) were discharged and 17% (33) died. There were missing data for 25.8% (51) of the patients for the lactate clearance, whereas 55% (108) patients had early lactate clearance and 19.7% (39) had delayed lactate clearance.On univariate analysis, mortality rate was higher in patients with delayed lactate clearance (38.4% vs 16.6%) and patients were 3.12 times (OR = 3.12; [95% CI: 1.37-7.09]) more likely to die as compared with early lactate clearance. Patients with delayed lactate clearance had higher organ dysfunction (79.4% vs 60.1%) and were 2.56 (OR = 2.56; [95% CI: 1.07-6.13]) times likely to have organ dysfunction. On multivariate analysis, after adjusting for age and co-morbids, patients with delayed lactate clearance were 8 times more likely to die than patients with early lactate clearance [aOR = 7.67; 95% CI:1.11-53.26], however, there was no statistically significant association between delayed lactate clearance [aOR = 2.18; 95% CI: 0.87-5.49)] and organ dysfunction.

**Conclusion:**

Lactate clearance is a better determinant of sepsis and septic shock effective management. Early lactate clearance is related to better outcomes in septic patients.

## Introduction

Sepsis is organ dysfunction syndrome secondary to dysregulated host immune response to infection [1]. Septic shock is defined as sepsis with refractory hypotension needing vasopressors support to keep the mean arterial pressure above 65mm Hg and serum lactate level above 2 mmol/l despite adequate volume resuscitation. The approximate burden of sepsis in 2017 was 48.9 million new cases and almost 11 million deaths worldwide [2], this increase is twice the previous numbers and the notable increase noted is due to the addition of data from middle and low socioeconomic countries [2,3], however, the prevalence in low and middle-income countries is still understudied [3]. In a study conducted in 16 Asian countries across 150 intensive care units; more than 28% of all admission in participating units from Pakistan had sepsis [4].

The high mortality related to sepsis has made extensive work happen as part of the efforts to understand the factors related to mortality. The gold standard test; blood cultures utility is limited as it takes a long time and is only 50% times positive in septic patients [5]. C-reactive protein and white blood cell count has also low yields while procalcitonin has better prognostic value but is not widely available [6]. Serum lactate level is of prime importance in the diagnosis and management of septic shock [7, 8, 9]. Serum lactate elevation is an important marker of impaired tissue perfusion in patients with septic shock and is often elevated even in the absence of arterial hypotension [10]. When oxygen delivery fails to meet tissue oxygen demand in critical illness, there is a compensatory increase in oxygen extraction. If the imbalance between oxygen delivery and consumption is uncorrected, the compensatory response is exhausted, resulting in oxygen debt, global tissue hypoxia, anaerobic metabolism, and lactate production [11].

High lactate level is known to be associated with high sepsis-related mortality [12]. The sensitivity and specificity of single lactate concentrations as markers of tissue hypoperfusion have been debated; however, serial measurements or lactate clearance over time may be better prognosticators of organ failure and mortality [13].

There is a lack of evidence in local literature regarding early lactate clearance in severe sepsis and septic shock. Single lactate measurement and serial lactate measurements have been reported to have prognostic significance. Therefore, the purpose of this study was to determine if early lactate clearance is associated with improved survival in patients admitted with sepsis in this part of the world.

## Materials and methods

This prospective cohort study was conducted from September 2019 to February 2020 at the Medicine Department of The Aga Khan University Hospital, Karachi. Patients were enrolled using consecutive sampling methods and based on their lactate clearance status (early vs. late) and followed for survival. Patients older than 18 year and sepsis were included. Patients having a myocardial infarction, pulmonary edema, hemorrhagic shock, trauma, seizure, pregnancy, or requiring immediate surgery were excluded.

**Sepsis** was defined as per the surviving sepsis guidelines [7].

Sepsis: suspected infection in the presence of SIRS

Systemic Inflammatory response syndrome (SIRS) criteria:

- Temperature >38°C or <36°C,
- Heart rate >90 beats/min,
- Respiratory rate >20 breaths/min, or PaCO2 <32 mm Hg, or
- White blood cell count >12,000 cells per mm3 < 4,000 cells per mm3, or >10% band cells.

**Severe Sepsis**: sepsis plus at least one organ dysfunction on admission.

**Septic Shock**: systolic blood pressure < 90 mm Hg after a 20 mL/kg fluid challenge.

**Elevated Lactate**: >4mmol/liter

**Early Lactate Clearance**: defined using the following formula: initial lactate at presentation (hour 0) minus lactate (hour 6), divided by initial lactate at presentation, and then multiplied by 100. A positive value denotes a decrease or clearance of lactate, whereas a negative value denotes an increase in lactate after 6 hours.

(a) lactate clearance: repeat lactate decreases by 10% or greater from the initial (or both initial and repeat levels <=2.0 mmol/L), and (b) lactate non-clearance: repeat lactate decreases by less than 10% from initial.

The proportion of organ dysfunction and deaths at the end of hospital stay in early lactate clearance vs. late lactate clearance groups was determined.

A minimum sample size of 180 (90 in each exposure group of lactate clearance percentage) was required to achieve 80% power considering the range of survival between 19% to 71% for the lactate clearance categories with a minimum difference of 28.5% in survival proportion between the two categories and 5% level of significance. This sample size was calculated using NCSS-Pass software. All patients received sepsis bundle management i.e broad-spectrum antibiotics, crystalloid for hypotension and vasopressors where appropriate.

### Data Collection

Data were collected via file reviews for six months from the date of getting exemption from the ethical review committee (ref- 2019-1589-4058) following variables, were considered: demographics, the suspected source of infection, vital signs, initial and repeat serum lactate concentrations, initially at admission and then 6 hours later, use of vasopressin, and intravenous fluid, information on comorbid conditions, hospital length of stay and outcome information. Illness severity was defined using the presence of individual organ system failures as assessed by the worst recorded values for each organ system: cardiovascular = initial Systolic BP less than 90 mmHg; pulmonary = new oxygen requirement or PaO2/FIO2 less than 300; renal = serum creatinine more than 2.0 mg/dL; hepatic = serum bilirubin greater than 2.0 mg/dL; hematologic = platelets less than 100,000/2L or international normalized ratio more than 1.5 sec; neurologic = a new finding of altered mental status (history or physical exam) and calculation of the Sequential Organ Failure Assessment (SOFA) score.

### Statistical methods

To describe the characteristics of the study population, we reported frequencies and proportions, SPSS 23.0 was used to analyze the data. More specifically, we reported frequencies and proportions to describe the characteristics of the study population for the categorical variables such as gender, presence of diabetes or hypertension, etc. We checked the normality assumption for continuous variables by histograms superimposed with the normal curve. We used a Chi-squared test or Fisher’s exact to assess the frequency distribution and the relationship between covariates and survival or organ dysfunction for categorical variables. We performed univariable logistic regression analysis to determine the individual effect of each significant predictor on survival or organ dysfunction. We considered a p-value of less than 0.05 for significant results. Finally, we conducted a multivariable logistic regression analysis to determine the adjusted effect of each determinant on survival or organ dysfunction. We presented the results of regression analysis by crude/unadjusted Odds ratio (OR) and adjusted odds ratio (AOR) with 95% Confidence Intervals (CIs).

## Results

### Descriptive characteristics of patients admitted to the hospital

A total 198 patients were included in the study. The study findings illustrated that almost half of the patients (45.54%) were older than 60 years and a quarter (25.75%) were young of 20 to 40 years old as sown in Table 1. The ratio of males and females was almost one to one with 51% being male and 49% females. A little over a quarter (27.8%) of the patients were diabetic, almost one-third (35.4%) were hypertensive, 14.6% had chronic kidney disease, 18.2% had ischemic heart disease, 19.7% suffered from lung disease, and 5.1% had chronic liver disease. Almost all of the admitted patients (84.3%) had undergone one or other surgical or therapeutic procedures during a hospital stay and one quarter (25.8%) stayed for more than a week in the hospital. A greater proportion of the patients (83.3%) had a platelet count of more than 100,000 and 11.1% had a previous history of blood transfusion (Table 1). Around 83% of the patients (n= 165) were discharged from the hospital and 17% of the patients died (n=33). Pneumonia was the most common cause of sepsis in 48.9%(97) (Table-2). There were missing data for 25.8% of the patients for the lactate clearance, whereas 55% of the patients had early lactate clearance and 19.7% had delayed lactate clearance. On the other hand, 18.69% (37) of the patients had multiple organ dysfunction, 47.47%(94) had single organ dysfunction, and 33.84%(67) had no organ dysfunction.

**Table 1:** Sociodemographic and clinical characteristics of the patients admitted in a tertiary care hospital (n=198)

Variables	Frequency (n)	Percentage (%)
Age (Years)		
20 to 40 Years	51	25.7
41 to 60 years	57	28.7
≥ 60 years	90	45.45
Gender		
Male	101	51
Female	97	49
Hypertension		
Yes	70	35.4
No	128	64.6
Diabetes Mellitus		
Yes	55	27.8
No	143	72.2
Chronic Kidney Disease		
Yes	29	14.6
No	169	85.4
Ischemic Heart Disease		
Yes	36	18.2
No	162	81.8
Lung Disease		
Yes	39	19.7
No	159	80.3
Chronic Liver Disease		
Yes	10	5.1
No	188	94.9
Any Procedures done		
Yes	167	84.3
No	31	15.7
Length of stay		
≤ 7 days	147	74.2
> 7 days	51	25.8
Platelets		
>100000	165	83.3
Less than 100000	33	16.7
Blood Transfusion		
Yes	22	11.1
No	176	88.9
Glasgow Coma Scale		
Mild (14-15)	151	76.3
Moderate (9-13)	38	19.2
Severe (3-8)	9	4.5

**Table 2:** Source of sepsis and organisms grown in cultures

Variables	Frequency (n)	Percentage (%)
Sepsis source		
Pneumonia	97	48.9
Viral fever/Dengue Fever	33	16.7
UTI/Pyelonephritis	32	16.2
Enteric fever	9	4.5
Meningitis	8	4
Infected wound/Bedsore	8	4
Spontaneous bacterial	5	2.5
peritonitis		
Malaria	3	1.5
Viral hepatitis	3	1.5
Blood cultures		
E.coli	10	5.10
Salmonella typhi	9	4.5
Klebsiella	5	2.5
Candida auris	2	1.1
Proteus	1	0.5
Staph aureus	1	0.5
Urine cultures		
E.coli	9	4.5
Klebsiella	4	2
Candida	3	1.5
Tracheal cultures		
Klebsiella	8	4
Acinetobacter	6	3
Staph aureus	5	2.5
E.coli	2	1.1
Pseudomonas	2	1.1
Candida	1	0.5
Pus cultures		
E.coli	5	2.5
Staph aureus	3	1.5
Streptococcus	1	0.5

### Characteristics of patients by mortality

Table 3 describes the characteristics of the patients by survival or mortality. We found that a higher proportion of the patients who died (57.6%) were older than 60 years old when compared with 43.0% of the patients discharged from the hospital. Similarly, 54.5% of the patients died and 22.4% of their counterparts were diabetic (p-value: <0.001). A significantly higher proportion of patients who died (60.6%) were hypertensive compared with patients discharged from the hospital (30.3%; p-value: 0.001). Mortality rate was higher in patients with delayed lactate clearance 38.4% (18/39) as compared to 16.6% (18/108) in patients with early clearance. About 45.5% of the patients who died had delayed lactate clearance when compared with 21.1% of patients discharged (p-value: 0.005) as shown in Table 3. Around 39.4% of the patients who died had undergone any procedure done as opposed to 10.9 % of the patients who were discharged from the hospital. A significantly higher proportion of the patients (63.6%) who died suffered from chronic kidney disease as opposed to 10.3% of the patients discharged. However, there were no significant differences between patients who died and those who were discharged by blood transfusion and gender.

**Table 3:** Sociodemographic and clinical characteristics of the patients by mortality and organ dysfunction

	Mortality					Organ Dysfunction						
Variables	No		Yes		P-Value	No organ dysfunction		Single organ dysfunction		Multi organ dysfunction		P-Value
	n	%	n	%		n	%	n	%	n	%	
Age (Years)												
20 to 40 Years	54	29.1	3	9.1		21	31.3	23	24.5	7	18.9	
41 to 60 years	46	27.9	11	33.3	0.054	18	26.9	27	28.7	12	32.4	0.71
≥ 60 years	71	43	19	57.6		28	41.8	44	46.8	18	48.6	

Gender												
Male	85	51.5	16	48.5	0.75	35	52.2	49	52.1	17	45.9	0.79
Female	80	48.5	17	51.5		32	47.8	45	47.9	20	54.1	

Lactate Clearance												
Early	90	78.9	18	54.5	**0.005**	43	84.3	47	68.1	18	66.7	0.09
Delayed	24	21.1	15	45.5		8	15.7	22	31.9	9	33.3	

Diabetes Mellitus												
No	128	77.6	15	45.5	**<0.001**	48	71.6	67	71.3	28	75.7	0.41
Yes	37	22.4	18	54.5		19	28.4	27	28.7	9	24.3	

Hypertension												
No	115	69.7	13	39.4		41	61.2	66	70.2	21	56.8	
Yes	50	30.3	20	60.6	**0.001**	26	38.8	28	29.8	16	43.2	0.26

Ischemic Heart Disease												
No	140	84.8	22	66.7	**0.013**	58	86.6	73	77.7	31	83.8	0.33
Yes	25	15.2	11	33.3		9	13.4	21	22.3	6	16.2	

Chronic kidney disease												
No	148	89.7	21	36.4	**<0.001**	62	92.5	79	84	28	75.7	0.059
Yes	17	10.3	12	63.6		5	7.5	15	16	9	24.3	

Chronic Liver Disease												
No	159	96.4	29	87.9		67	100	87	92.6	34	91.9	
Yes	6	3.6	4	12.1	**0.04**	0	0	7	7.4	3	8.1	0.06

Lung Disease												
No	137	83	22	66.7	**0.031**	60	89.6	72	76.6	27	73	**0.05**
Yes	28	17	11	33.3		7	10.4	22	23.4	10	27	

Any procedure done												
No	147	89.1	20	60.6	**<0.001**	58	86.6	83	88.3	26	70.3	**0.03**
Yes	18	10.9	13	39.4		9	13.4	11	11.7	11	29.7	

Length of Stay												
≤ 7 days	134	81.2	13	39.4		49	73.1	73	77.7	25	67.6	
> 7 days	31	18.8	20	60.6	**<0.001**	18	26.9	21	22.3	12	32.4	0.47

Platelets												
>100000	143	86.7	22	66.7	**0.005**	56	83.6	74	78.7	35	94.6	0.09
Less than 100000	22	13.3	11	33.3		11	16.4	20	21.3	2	5.4	

Blood Transfusion												
Yes	18	10.9	4	12.1	0.84	9	13.4	9	9.6	4	10.8	
No	147	89.1	29	87.9		58	86.6	85	90.4	33	89.2	0.74

Glasgow Coma Scale												
Mild (14-15)	144	87.3	7	21.2		54	80.6	73	77.7	24	64.9	
Moderate (9-13)	20	12.1	18	54.5	**<0.001**	11	16.4	19	20.2	8	21.6	**0.05**
Severe (3-8)	1	0.6	8	24.2		2	3	2	2.1	5	13.5	

*P-Values are generated using chi-squared or Fisher exact test and the bold ones are considered statistically significant

### Characteristics of patients by organ dysfunction

Table 3 describes the characteristics of the patients by organ dysfunction. We found that a higher proportion of the patients with multiple organ dysfunction (33.3%) had delayed lactate clearance when compared with 31.9% of the patients with single organ dysfunction and 15.7% of the patients who did not have any organ dysfunction at all (p-value: 0.09). Similarly, 24.3% of the patients with multiple organ dysfunction had chronic kidney disease when compared with 16.0 % of the patients with single organ dysfunction and 7.5% of the patients with no organ dysfunction at all (p-value: <0.059). A significantly higher proportion of patients with multiple organ dysfunction (27.0%) had lung disease when compared with 23.4 % of the patients with single organ dysfunction and 10.4% of the patients with not any organ dysfunction at all (p-value: 0.05). About 13.5% of the patients with multiple organ dysfunction had severe worsening of the score on the Glasgow coma scale when compared with 2.1% of patients with single organ dysfunction and 3% with no organ dysfunction (p-value: 0.05) as shown in Table 3. Around 29.7 of the patients with multiple organ dysfunction had undergone any procedure done as opposed to 13.4 % of the patients with no organ dysfunction. Whereas there were no significant differences between patients with and without organ dysfunction by blood transfusion, age, gender, ischemic heart disease, hypertension, and diabetes mellitus (Table 3).

### Factors associated with mortality: Findings of univariable logistic regression analysis

Table 4 shows the findings of factors associated with mortality with a special focus on the association between delayed lactate clearance and mortality. We found that patients with delayed lactate clearance were 3.12 times (OR = 3.12; [95% CI: 1.37-7.09]) more likely to die when compared to patients with early lactate clearance (Table 3). Likewise, patients who were diabetic were 4.15 times more likely to die when compared to the non-diabetic patients (OR = 4.15; [95% CI:1.91-9.03]). Similarly, hypertensive patients were 3.54 times more likely to die when compared to their counterparts (OR = 3.54; [95% CI:1.63-7.67]). Furthermore, patients with lung disease were 2.46 (OR = 2.46; [95% CI: 1.075.61]) times likely to die when compared to those without lung disease. Patients with ischemic heart disease were 2.80 (OR = 2.80; [95% CI: 1.21-6.48]) times more likely to die than patients without ischemic heart disease (Table 4). Similarly, patients with any procedure done were 5.31 (OR = 5.31; [95% CI: 2.26-12.45]) times likely to die when compared to those without any procedure done. Patients with prolonged stay and platelet count of less than 10,000 were 6.65 (OR = 6.65; [95% CI: 2.98-14.8]) and 3.25(OR = 3.25; [95% CI: 1.38-7.61]) times likely to die respectively as opposed to their counterparts (Table 4).

**Table 4:** Association between delayed lactate clearance and mortality and organ dysfunction in patients admitted in a tertiary care hospital (n=198)

	Risk factors for Mortality						Risk factors for Organ Dysfunction					
Variables	Crude			Adjusted Estimates			Crude			Adjusted Estimates		
	OR	95%CI		AOR	95%CI		OR	95%CI		AOR	95%CI	
Age (Years)												
20 to 40 Years	1			1			1					
41 to 60 years	3.83	1.01	14.59	29.19	0.21	41.5	1.52	0.68	3.34		NA	
≥ 60 years	4.28	1.2	15.27	2.77	0.02	33.7	1.55	0.76	3.16			

Lactate Clearance												
Early	1			1			1			1		
Delayed	3.12	1.37	7.09	7.67	1.11	53.26	2.56	1.07	6.13	2.18	0.87	5.49

Diabetes Mellitus												
No	1			1			1			1		
Yes	4.15	1.91	9.03	8.56	1.18	62.01	0.95	0.49	1.84	0.53	0.22	1.31

Hypertension												
No	1			1			1			1		
Yes	3.54	1.63	7.67	56.36	3.57	889.83	0.79	0.43	1.47	0.6	0.27	1.33

Chronic kidney disease												
No	1						1			1		
Yes	4.97	2.08	11.86		NA		1.01	2.78	7.66	6.13	1.72	21.86

Lung Disease												
No	1				NA		1			1		
Yes	2.46	1.07	5.61				1.15	2.77	6.67	2.61	0.97	6.99

Ischemic Heart Disease												
No	1			1			1				NA	
Yes	2.8	1.21	6.48	0.15	0.01	1.77	1.67	0.73	3.79			

Any procedure done												
No	1						1			1		
Yes	5.31	2.26	12.45		NA		1.3	0.56	3.01	1.41	0.55	3.63

Length of Stay												
≤ 7 days	1			1			1				NA	
> 7 days	6.65	2.98	14.8	2.59	0.53	12.7	0.91	0.47	1.79			

Platelets												
>100000	1			1			1				NA	
Less than 100000	3.25	1.38	7.61	305.74	17.9	5195.87	1.03	0.46	2.27			

Glasgow Coma Scale												
Mild (14-15)	1			1							NA	
Moderate (9-13)	18.51	6.87	49.84	134.86	11.17	1627.16	1					
Severe (3-8)	164.57	18.01	1504.6	1251.02	28.84	54260.43	1.36	0.63	2.97		NA	
CRP	0.98	0.97	0.99	0.97	0.94	1	1.95	0.39	9.71			

The model mortality is adjusted for age, DM, HTN, IHD, LOS, Platelet count, and Glasgow Coma Scale. The model for organ dysfunction is adjusted for DM, HTN, chronic kidney disease, lung disease, and any procedure done. OR: Odds ratio; NA: Not applicable; AOR: Adjusted Odds r.

### Factors associated with organ dysfunction: Findings of univariable logistic regression analysis

Table 4 shows the findings of factors associated with organ dysfunction with a special focus on the association between delayed lactate clearance and organ dysfunction. The study findings revealed that patients with delayed lactate clearance were 2.56 (OR = 2.56; [95% CI: 1.07-6.13]) times likely to have organ dysfunction when compared to patients with early lactate clearance. Similarly, patients with chronic kidney disease were 2.78 (OR = 2.78; [95% CI: 1.01-7.66]) times more likely to have organ dysfunction than patients without chronic kidney disease (Table 4). We did not find any significant differences between patients with and without organ dysfunction on factors such as age, gender, hypertension, any procedure is done, lung disease, platelet count, and length of stay as shown in table 4.

### Association of delayed lactate clearance with mortality: Findings of multivariable analysis

The results of the multivariable analysis were adjusted for patient’s age, comorbid such as DM, HTN, and is-chemic heart disease, platelet count, and length of stay. The adjusted results demonstrated a positive relationship between delayed lactate clearance and mortality, meaning that after adjusting for potential confounders, patients with delayed lactate clearance were more than 8 times more likely to die than patients with early lactate clearance [aOR = 7.67; 95% CI:1.11-53.26)]. Other statistically significant factors predicted the mortality included DM (aOR = 8.56; [95% CI :1.18-62.01]), HTN (AOR = 56.36; [95% CI :3.57-889.83]), (aOR = 1.37; [95% CI (0.52-3.66]) and platelet count as well as patient’s level of consciousness as shown in Table 4.

### Association of delayed lactate clearance and organ dysfunction: Findings of multivariable analysis

The results of the multivariable analysis were adjusted for the patient’s comorbidities. The results demonstrated that there was no statistically significant association between delayed lactate clearance [aOR = 2.18; 95% CI: 0.87-5.49)] and organ dysfunction in the final adjusted model after controlling for potential confounders. However, there was a statistically significant association between chronic kidney disease (aOR = 6.13; [95% CI:1.72-21.86]) and organ dysfunction after controlling for potential confounders persisted in the adjusted model, however, 95% CI for these associations were not precise as shown in Table 4.

## Discussion

Our study illustrated that delayed lactate clearance is strongly associated with mortality in septic patients along with diabetes mellitus, hypertension, and low platelet count. A retrospective multicenter study across 335 ICU in USA by Burno RR, et al. and colleagues showed six hours lactate clearance to be of high prognostic value, delayed clearance (<10% at six hours) was associated with higher sepsis-related organ dysfunction and higher intensive care mortality that is 32% vs 14% compared to the early lactate clearance group [14]. Nguyen HB, et al. noted early lactate clearance with improved outcomes in sepsis and septic shock patients [15]. Innocenti, et al. from Italy found the same results in 268 septic patients in whom more than 10% lactate clearance at 6 hours was associated with better survival [16]. Taniguchi et al. noted procalcitonin and lactate clearance as an exponent of improving severity in septic shock secondary to the abdominal source [17]. Another study from Japan revealed that enhanced lactate clearance during the first 24 hours was notably linked with reduced 28 days mortality in septic shock patients with bilirubin less than 2 mg/dl, however, lactate clearance was not related to decreasing mortality in patients whose total bilirubin was more than 2 mg/ dl [18]. In contrary to our finding, Moustafa et al. from Egypt did not find the relation between lactate clearance and mortality [19].

We could not establish organ dysfunction in septic patients to be statistically significantly related to delayed lactate clearance, however, patients having multiple organs dysfunction did have delayed lactate clearance as compared to the patients who did not have any organ dysfunction. Scott et al. depicted that lactate normalization was related to less risk of persistent organ dysfunction, but lactate clearance did not have an impact on organ dysfunction [19]. Organ dysfunction results from dysregulated host immune response to sepsis, triggering macro and microvascular failure leading to a state of global hypoperfusion and vital organ dysfunction [20].

We also found diabetes mellitus, in septic patients to be positively correlated with mortality. Meregalli A, et al. depicted that diabetics have not only increased vulnerability to infection but worse outcomes as well [21]. Alteration in the function of neutrophils, macrophages, natural killer cells, CD4 T cells, and CD8 T cells and abnormal antibodies response are the possible reasons in diabetic patients for increased susceptibility to infections [22]. On the contrary, Vught LA et al. demonstrated that diabetes mellitus was not related to higher 90-days mortality in septic patients, however, hypoglycemia in diabetic patients and hyperglycemia in non-diabetic patients were responsible for higher mortality in the same cohort [23].

Hypertension was the other disorder responsible for higher sepsis-related mortality in our study cohort. Hypertension increases the risk of sepsis [24], and hypertensive patients on antihypertensive medication have lower sepsis-related mortality as compared to a hypertensive individual who is not on antihypertensive medications [24]. Dial S, et al. found that patients treated with angiotensin receptor blockers don’t seem to be at higher risk of sepsis and sepsis related mortality and renal failure, however, hypertensive patients on angiotensin-converting enzyme inhibitors have higher sepsis-related mortality [25]. The plausible explanation is that sepsis results in renin-angiotensin-aldosterone system activation, angiotensin II production which is an inflammatory agent and is associated with organ failure and mortality [26]. We could not study the impact of different classes of antihypertensive medications on sepsis outcomes.

Among laboratory parameters we observed low platelets count to be related to higher sepsis-related mortality. Semeraro F, et al. showed that septic patients who had thrombocytopenia on day one or developed it within 28 days of illness had more severe disease and higher mortality as compared to those with no thrombocytopenia [27]. Thrombocytopenia was found to be directly related to disease severity and lactate level [28]. It was found to be related to the risk of major bleed, organ failure, and higher mortality and non-resolving thrombocytopenia is even more strongly related to higher mortality [28].

Our study findings will be valuable in managing septic patients. Aiming for Lactate clearance instead of checking single lactate levels will be more meaningful in terms of decreasing mortality in septic patients. Multicenter and higher sample-sized studies are needed to substantiate our findings.

## Conclusion

Lactate clearance is a better determinant of sepsis and septic shock effective management. Early lactate clearance is related to better outcomes in septic patients.
